# Rearticulating the promotion of Japanese language and culture from the perspective of “soft power”: planning and effects

**DOI:** 10.3389/fpsyg.2024.1371271

**Published:** 2024-05-30

**Authors:** Jingshu Gu, Shiping Deng

**Affiliations:** ^1^School of Foreign Languages and Literature, Suzhou University of Science and Technology, Suzhou, China; ^2^Department of Applied Foreign Language Studies, Nanjing University, Nanjing, China

**Keywords:** Japan, soft power, language promotion, cultural communication, language education planning, prestige planning of language and culture

## Abstract

The concept of soft power has engendered lively discourse within the international community. The development of a nation’s soft power frequently hinges on cultural communication and the promotion of language. This manuscript concentrates on Japan as a case study and undertakes an investigation of the methods it has employed to cultivate its soft power. To accomplish this objective, Japan’s strategies for cultural communication and the promotion of the Japanese language are comprehensively examined. From the perspective of language planning, prestige planning of language and culture constitutes a crucial mode of cultural communication, whereas language education planning (or acquisition planning) is the primary method of language promotion. Japan has adeptly disseminated its culture overseas through cultural communication and language promotion. On one hand, it fashions a “cool culture” embodied by anime and manga to augment cultural appeal and amplify national image. On the other hand, it advances the Japanese language abroad by establishing supportive institutions, dispatching experts and volunteers, and creating online teaching materials. Building on Japan, this paper establishes a theoretical framework for the construction of soft power, employing the aforementioned two approaches as valuable guides for research on soft power.

## Introduction

After the Cold War, numerous nations, including Japan, began expanding their international influence by reinforcing their soft power. In recent years, researchers have become increasingly interested in soft power studies and have generated abundant findings (Nye, 1990; Nye, 2004; Roselle et al., 2014), enabling us to gain a more profound comprehension of the intrinsic nature of soft power and how it may be augmented from diverse perspectives. However, the role of language in the construction of soft power remains insufficiently explored (Hashimoto, 2018). The present study took the initiative to probe into the case of Japan to analyze how language promotion and cultural communication can improve a country’s soft power within the framework of language planning, with the goal of providing a new perspective for research on soft power.

Japan has been dedicated to augment its soft power by investing heavily in the realm of culture for numerous years. Since the 1990s, Japan has incorporated cultural communication and language promotion into its national policy of “cultural diplomacy” as part of efforts to improve its international status. Cultural diplomacy refers to a national foreign policy introduced by the Ministry of Foreign Affairs of Japan during the 1990s, which was reaffirmed in December 2019 with the aim of strengthening the promotion of Japanese language education overseas. Japan has employed a multicultural communication strategy that includes both popular and traditional culture (Hashimoto, 2018) to elevate its international image, while concurrently developing language education planning encompassing teacher development, language testing, and teaching resources to promote the Japanese language abroad, thus enhancing its international influence. These two approaches have enabled Japan to consistently rank among the world’s foremost players in the domain of soft power, as evidenced by its ranking of 4th in 2020, 2nd in 2021, and 5th in 2022 in the Global Soft Power Index by Brand Finance. Therefore, Japan can be considered a representative case for investigating the role of language in the construction of soft power.

### Research context

Japan’s soft power development adopts a “three-in-one” model, comprising the Agency for Cultural Affairs, the Japan Foundation, and various private associations (refer to Table 1). The Agency for Cultural Affairs is responsible for cultural policy-making and overseeing several independent corporate bodies aimed at cultural development. The Japan Foundation implements comprehensive international cultural exchange programs and is mainly responsible for overseas Japanese language education. Private organizations like the Association for the Promotion of Japanese Language Education assist in promoting Japanese language education. This article will incorporate the three aspects mentioned, but its primary focus will be on the official reports and data from the Japan Foundation, which is the key sector and main implementing agency for soft power development in Japan.

**Table 1:** Implementation organization and division of labor of Japan’s soft power strategy.

Implementation organization	Division of labor
Agency for Cultural Affairs	Cultural heritage protection policies
	Cultural arts revitalization policies
	Media/cultural industry promotion strategies
The Japan Foundation	International cultural arts exchanges
	Overseas Japanese language education
	Japan-related research and knowledge exchanges
Association for the Promotion of Japanese Language Education	Approval and accreditation of Japanese Language education institutions; supervision of the legality of Japanese language education institutions; research, study and development of Japanese language education; promotion of cooperation between Japanese language education institutions and universities, etc.
Japan Educational Exchanges and Services	International student welfare, communication, language proficiency tests, etc.
Association for Japanese-Language Teaching	To support Japanese language teaching and create various Japanese language learning programs
Association for Japanese Language Education	Japanese language education and academic exchanges

### Literature review

Research on soft power has its origins in Western politics and economics. Since the 1870s, scholars in the field of politics have generally focused on the “material” and “immaterial” dimensions of state power (Morgenthau, 1946). Concurrently, American economist Lawrence R. Klein proposed the “comprehensive national power equation,” which divides comprehensive national power into “material power” and “spiritual power” based on quantitative comparison and analysis. It is believed that these two components can interact with each other to jointly strengthen a country’s comprehensive national power (Cline, 1975). All of these studies suggest that achieving national strategic goals is highly intricate and contains many intangible factors that are difficult to measure using static criteria.

The attention paid to intangible forces in the domains of economics and politics has led researchers to consider the “cultural force” of state power. Following the end of the Cold War, the United States emerged as the sole superpower in the world, leading to a paradigm shift in the global order. Against this backdrop, building on the work of previous scholars, American scholar Joseph Nye introduced the concept of “soft power,” which emphasizes non-coercive means of achieving national objectives. In contrast to hard power, which involves tangible factors such as gross domestic product, military strength, and natural resources, soft power centers on intangible factors like national cohesion, cultural identity, and participation in international institutions (Nye, 1990, 2004). Soft power derives from a country’s culture, values, and international norms, with culture being its core (Nye, 2011). Soft power does not rely on force or coercion, but rather on attraction and persuasion to achieve national goals (Lukes, 2005).

The aforementioned studies underscore that soft power entails the potential and appeal derived from a country’s culture, values, and social institutions, which is in line with the essence of “cultural diplomacy” in politics. Cultural diplomacy typically involves a protracted process of achieving mutual understanding and recognition between nations through the presentation of their respective cultures to one another (Espinosa, 1977; Finn, 2003; Gienow-Hecht and Donfried, 2010; Ang et al., 2015). Cultural diplomacy is chiefly undertaken by national governments to propagate their political system, values, and cultural traits to other nations in order to establish, enhance, and sustain relations with them, thereby accomplishing national strategic objectives (Signitzer, 2013; Henne, 2022). Such cultural communication serves to cultivate a country’s soft power. Effective cultural diplomacy involves not only promoting a country’s culture but also its language, as language plays a crucial role in both culture and communication with political implications (Paschalidis, 2009). Language is not only a means of conveying information, but also is the carrier of culture and has the power to evoke emotional resonance, making language promotion an essential aspect that contributes significantly to the development of a nation’s soft power.

The above mentioned two aspects, cultural communication and language promotion, which are interconnected with each other, constitute vital sources of soft power (Iskhakova, 2021; Repnikova, 2022). Language planning, a multifaceted endeavor that includes status, corpus, education (or acquisition), and prestige planning (Haugen, 1983; Cooper, 1989; Haarmann, 1990), exerts a significant influence on both the dissemination of culture and the promotion of language. In particular, language education planning holds substantial sway in advancing a language, while language prestige planning may amplify the allure of a culture (Noack, 2022). Thus, both language education and prestige planning are instrumental in the construction of national soft power. Nevertheless, only a few studies (Gil, 2017; Hashimoto, 2018) address the issue of language planning in the context of soft power development. The full potential of language in augmenting a nation’s soft power still remains to be thoroughly investigated. In fact, while the majority of contemporary research on soft power neglects the significance of language planning, studies on language education planning and language prestige planning also overlook the implications of national soft power. Consequently, it is imperative to devise a comprehensive framework grounded in specific case studies to enhance our comprehension of the intricate relationship between language planning and national soft power.

## Research design

### Research question

This paper aims to examine the strategies for building soft power using Japan as a case study. “Cultural diplomacy” is considered the principal diplomatic strategy and guiding principle of soft power construction in Japan. Two main approaches to effectively build soft power in Japan in the context of cultural diplomacy are through language promotion and cultural communication (Henne, 2022). In general, this paper will address the following two research questions:

1. What roles do language education planning and language prestige planning play in language promotion and cultural communication so as to build Japan’s soft power?
2. How do language education planning and language prestige planning work together to facilitate the interaction between cultural communication and language promotion, thereby enhancing Japan’s soft power?

### Conceptual framework

**Figure 1:**
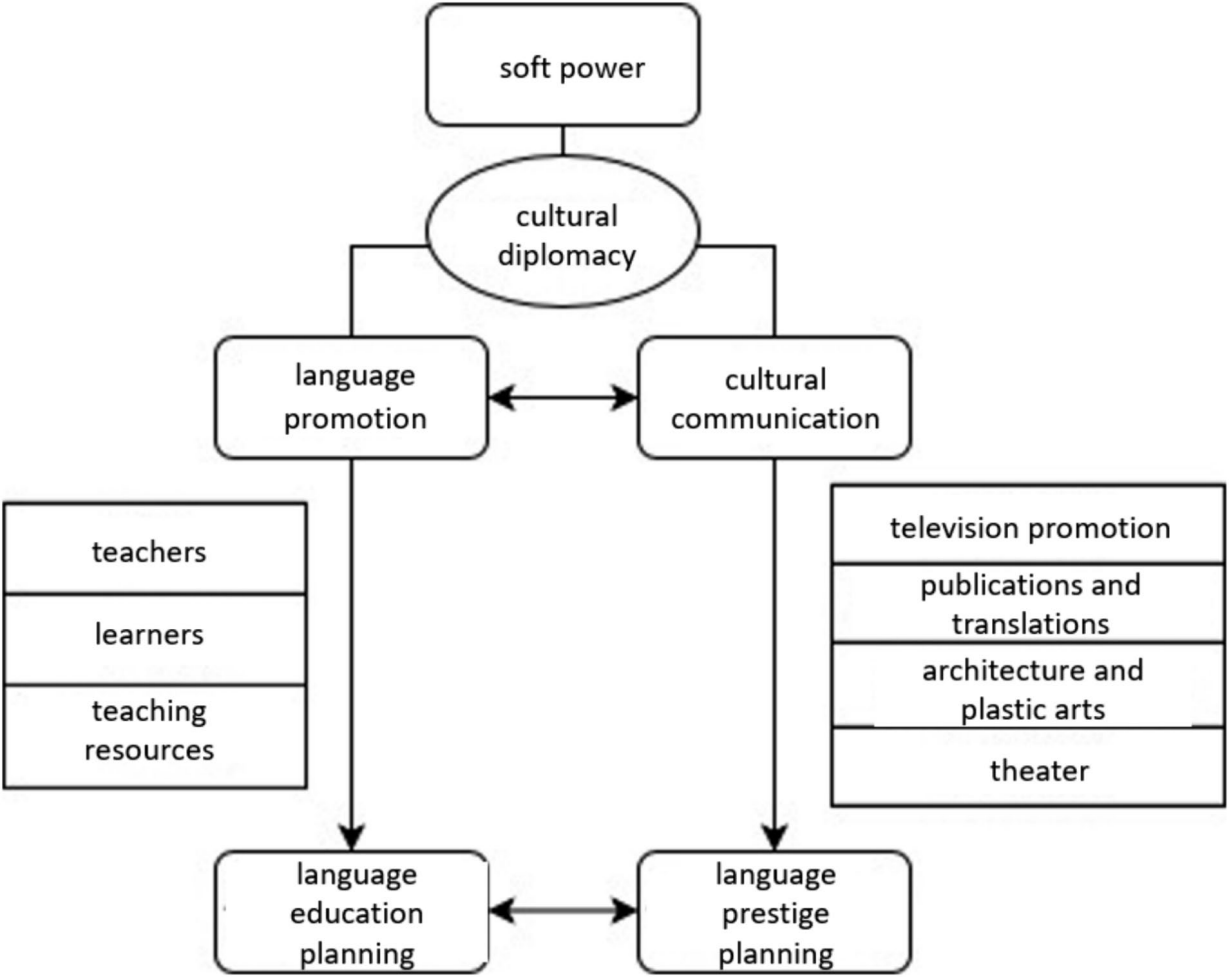
Conceptual framework of this study.

Previous studies have proved the interconnectedness between language education planning and language prestige planning (Baldauf, 2006). Based on that, this paper, then, is to prove and clarify how language and culture work together to enhance national soft power, as illustrated in the conceptual framework presented in Figure 1. The two approaches are mutually reinforcing, with language promotion focusing on teachers, learners, and teaching resources, and cultural communication highlighting various forms of media such as film, television, publications, translations, architecture, and theater. From a language planning perspective, all these activities fall under the purview of language education planning and language prestige planning, which can increase the effectiveness of cultural diplomacy in building soft power. Furthermore, this study utilizes the global program evaluation system proposed by Held et al. (2000) to evaluate programs on cultural communication and language promotion. This system mainly considers the program’s (1) extension, or the geographical coverage of the global program, (2) intensity, or the number of global programs, (3) velocity, or the speed at which the global project occurs, and (4) impact, or the consequences or outcomes of the global project.

### Data sources and scope of examination

This paper examines Japanese language promotion over the span of 2012–2021 (10 years in total). Four main sources of data on the Japan Foundation’s overseas operations are available: annual reports, self-assessment of its business performance, triennial surveys of Japanese language education overseas, annual performance evaluation reports by the Independent Administrative Institution Evaluation Committee of the Ministry of Foreign Affairs of Japan.

In order to keep this paper up to date, the analysis mainly focuses on the Japan Foundation’s annual reports (numbers 1–9) and the self-assessment report for 2021 (number 10), while other reports are used as references (numbers 11–14). All cases and data mentioned below are from Table 2. For example, “number 2” refers to the data or case from “The Japan Foundation 2013 Annual Report.”

**Table 2:** Data sources for this study.

Number	Report name	Publisher	Survey time	Year of issue
1	2012 Annual Report	The Japan Foundation	2012	2013
2	2013 Annual Report	The Japan Foundation	2013	2014
3	2014 Annual Report	The Japan Foundation	2014	2015
4	2015 Annual Report	The Japan Foundation	2015	2016
5	2016 Annual Report	The Japan Foundation	2016	2017
6	2017 Annual Report	The Japan Foundation	2017	2018
7	2018 Annual Report	The Japan Foundation	2018	2019
8	2019 Annual Report	The Japan Foundation	2019	2020
9	2020 Annual Report	The Japan Foundation	2020	2021
10	2021 Performance Report (Self-Assessment Report)	The Japan Foundation	2021	2022
11	Survey Report On Japanese-Language Education Abroad 2009	The Japan Foundation	2009–2010	2011
12	Survey Report On Japanese-Language Education Abroad 2012	Kurosio Publishers	2012	2013
13	Survey Report On Japanese-Language Education Abroad 2015	The Japan Foundation	2015–2016	2017
14	Survey Report On Japanese-Language Education Abroad 2018	The Japan Foundation	2018–2019	2020

### Findings

Based on the main responsibilities of the Japan Foundation, Japanese language promotion and cultural communication, this paper makes a deep analysis of the data in Table 2 quantitatively and then performs qualitative analysis in accordance with the reports. The details are as follows.

### Japanese language promotion

The Japan Foundation adopts an overarching strategy of dividing target regions into specific geographic areas, with a limited number of major countries in each continent or region being designated as hubs for Japanese language promotion. These hubs prioritize the establishment of teacher training platforms to exert influence on neighboring areas and expand the scope of promotion. In this paper, the analysis focuses on data from 2012 and 2015 (designated as numbers 2 and 3, respectively) to comprehend the general status of overseas Japanese language education planning, with an exclusion of uncertain external factors such as the epidemic. The investigation encompasses three indices: Japanese language education institutions, Japanese language teachers, and the number of Japanese language learners. According to the findings, China’s mainland is home to the highest number of Japanese language learners, followed by Indonesia, South Korea, Australia, China’s Taiwan region, and Thailand. In terms of the number of Japanese language education institutions, South Korea has the most, followed by Indonesia and China, and for the number of Japanese language teachers, China leads, followed by South Korea and Indonesia (refer to Table 3). From a macro perspective, Japan directs greater efforts toward Japanese language education and promotion in its Asian neighbors, highlighting that the comprehensive layout of its soft power building revolves around Asia as the axis and extends to the surrounding areas.

**Table 3:** Japanese language education institutions, Japanese language teachers and Japanese language learners in 2015 (by top 15 countries/regions in terms of number of learners).

Ranking	Country/Region	Japanese language education institutions 2011		Japanese language teachers 2013		Japanese language learners 2015	
		Quantity	Rise (%)	Quantity	Rise (%)	Quantity	Rise (%)
1	China	2,115	+17.5	18,312	+9.3	953,283	−8.9
2	Indonesia	2,496	+6.4	4,540	+0.0	745,125	−14.6
3	Korea	2,862	−26.9	14,855	−16.6	556,237	−33.8
4	Australia	1,643	+17.3	2,800	+4.3	357,348	+20.5
5	Taiwan (Region)	851	+9.9	3,877	+9.4	220,045	−5.7
6	Thailand	606	+30.3	1911	+37.8	173,817	+34.1
7	United States	1,462	+0.9	3,894	−8.8	170,998	+9.7
8	Vietnam	219	+21.7	1795	+17.5	64,863	+38.7
9	Philippines	209	+18.1	721	+29.7	50,038	+54.4
10	Malaysia	176	−10.2	430	−15.5	33,224	+0.4
11	New Zealand	257	−8.5	378	−12.3	29,925	−0.4
12	India	184	−9.8	655	+13.9	24,011	+19.4
13	Brazil	352	+8.3	1,140	+0.7	22,993	+15.5
14	Hong Kong (Region)	70	−4.1	523	−15.4	22,613	+0.3
15	France	222	+8.3	723	+3.1	20,875	+8.1
Total		16,179	+0.8	64,108	+0.5	3,655,024	−8.3

### Japanese language teacher development

The bedrock of promoting language is language instruction, which naturally requires skilled educators. To support Japanese language education in target countries and expand linguistic influence, the Japan Foundation places high importance on investing in Japanese language teacher training, increasing the number of Japanese language teachers, and aiding Japanese language education institutions. Concrete measures, such as developing a platform for teacher advancement and integrating teacher development with institutional growth, are implemented to expand teacher education and training in regions where Japanese language instructors are situated. This effort culminates in a dissemination pattern from a central hub to its surrounding areas.

The Sakura Network is a teacher development support program that was established in 2007 by the Japan Foundation and Japanese language education institutions worldwide. Its main objective is to enhance the research and teaching abilities of educators, with the goal of expanding the influence of Japanese language and stabilizing faculty resources. Member institutions of the Sakura Network have access to various training programs and vocational training provided by the Japan Foundation, which can greatly facilitate the personal development of teachers belonging to those institutions. Additionally, the Foundation expects that Japanese language teachers will contribute significantly to Japanese language education in their respective countries or regions while experiencing personal growth. To support Japanese language education institutions, the Sakura Network provides financial subsidies for teacher salaries and helps them in procuring teaching materials and studying teaching methods.

**Figure 2:**
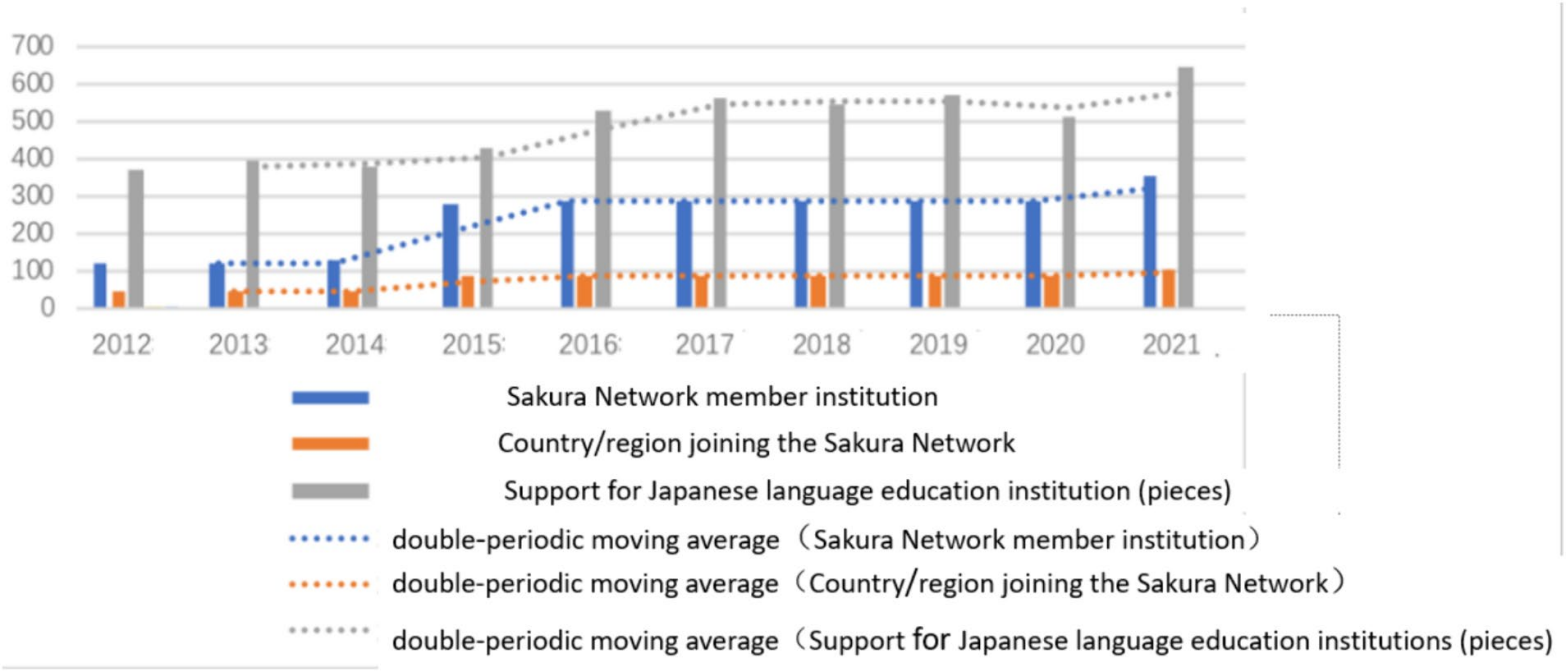
Japanese language teacher training, Sakura Network cultivation plan and support for Japanese language education institutions (2012–2021).

The data from Table 4 covering the period of 2012 to 2021 and the corresponding clustered column charts and moving average depicted in Figure 2 demonstrate a clear upward trend in the number of member institutions and countries or regions served by the Sakura Network, with a relatively stable rate of increase. Additionally, the number of instances in which the Japan Foundation provided aid to Japanese language education institutions has shown a marked increase, particularly in 2021. Notably, the peak number of cases coincides with the normalization of the pandemic, indicating that Japan has stepped up its support for Japanese language education institutions in the face of this unprecedented challenge.

**Table 4:** Japanese language teacher training, Sakura Network cultivation plan and support for Japanese language education institutions (2012–2021).

Year	Sakura Network member institution	Country/region joining the Sakura Network	Support for Japanese language education institutions (number of events)
2012	123	46	371
2013	126	47	395
2014	127	47	385
2015	284	92	435
2016	287	91	530
2017	288	92	568
2018	292	93	547
2019	292	93	569
2020	292	93	514
2021	357	102	652

The sourcing of teachers, their training and incentivization are all integral components of language education planning. The Sakura Network has substantially amplified the scope of Japanese language teacher training, extended avenues for the personal development of Japanese language teachers, and aligned with the framework of Japanese language education planning in terms of personnel policy. The inception of the Sakura Network highlights the comprehensiveness of the Japanese language promotion strategy, which extends from a singular focal point to encompass a larger area, and leverages the benefits of a collective effort for greater scale, thereby reflecting the progression of overseas Japanese language education planning.

### Japanese language education evaluation system: standards and tests

**Figure 3:**
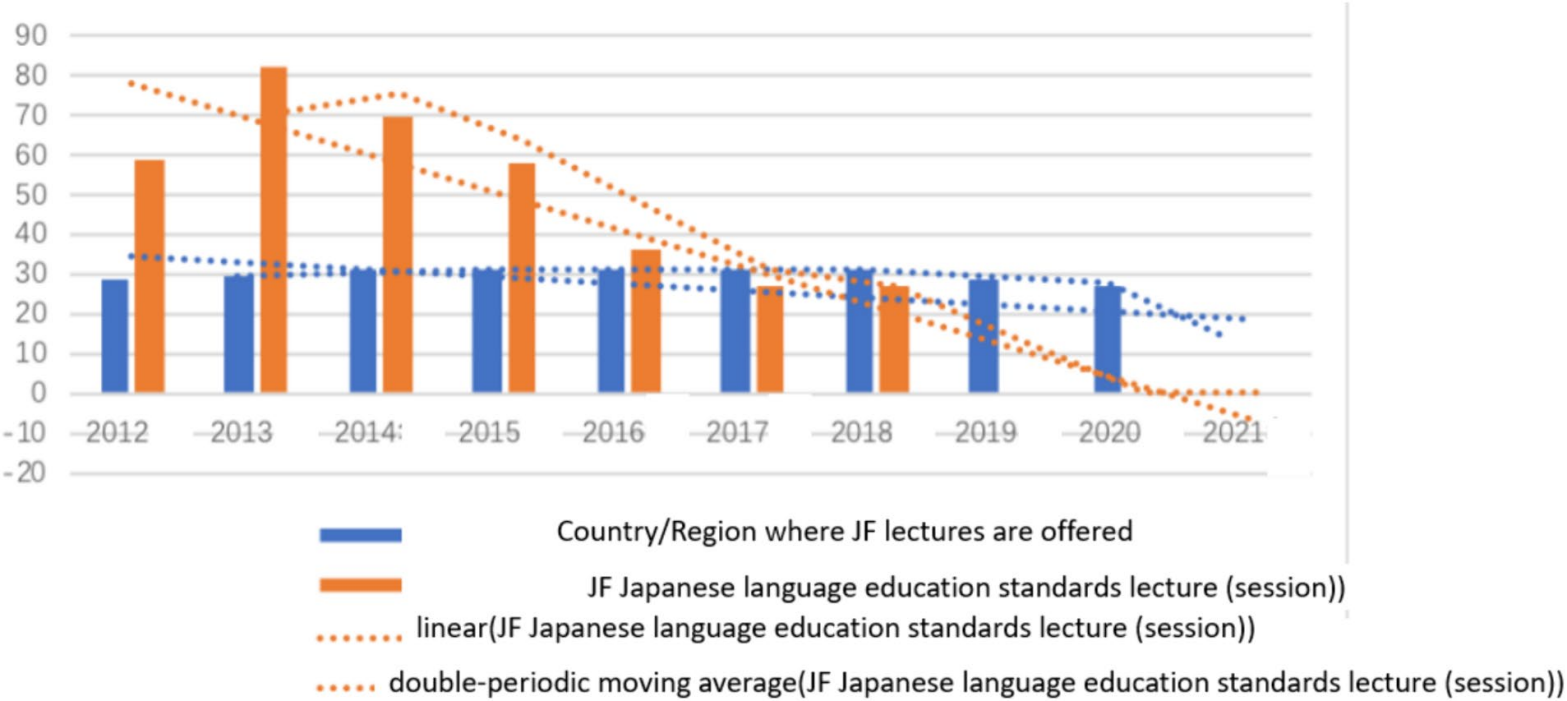
Promotion of JF Japanese education standards (2012–2021).

The Japan Foundation developed “The JF Japanese Language Education Standards” in 2010 as a means of assessing the Japanese language proficiency of non-native speakers based on the Common European Framework of Reference for Languages (CEFR). The Standards are designed to evaluate general language knowledge, operational skills, and communication skills of non-native speakers in everyday life and employment. They are widely employed in Japanese language teaching, textbook development, and teacher training. Statistical analysis of lecture attendance on the JF Japanese Language Education Standards from 2012 to 2018 reveals an initial rise followed by a subsequent decline. Since 2019, the Japan Foundation has shifted its promotional efforts from counting the number of lectures to monitoring the number of hits on the “JF Japanese Language Education Standards” website1, which registered about 245,000 and 268,000 hits in 2019 and 2020, respectively (Table 5). The figures for 2021 are not yet available. The promotion of the Standards has been actively pursued since its inception, with a shift in focus from offline to online in response to the trends and realities of online learning and work brought about by the pandemic. The development and dissemination of these Standards in various countries will help standardize Japanese language proficiency evaluation, facilitate Japanese language education, and foster a unified approach to Japanese language proficiency standards both in Japan and abroad (Figure 3).

**Table 5:** Promotion of JF Japanese Language Education Standards (2012–2021).

Year	Country/Region where JF lectures are offered	JF Japanese language education standards lecture (session)
2012	29	59
2013	30	82
2014	31	70
2015	31	58
2016	31	36
2017	31	27
2018	31	27
2019	29	245,000 hits
2020	27	268,000 hits
2021	—	—

As mentioned in the previous section, teacher growth, large-scale Japanese language teacher training and support for Japanese language education institutions are all important strategies of Japanese language promotion. In addition, the Japan Foundation also focuses on the promotion of standardized Japanese language examinations.

The Japanese-Language Proficiency Test (hereafter referred to as JLPT) is a standardized assessment of Japanese language proficiency jointly sponsored by the Japan Foundation and the Japan Educational Exchanges and Services since 1984. At its inception, only 7,000 candidates sat for the test, whereas today, more than 880,000 examinees worldwide have taken the exam, with test sites in 73 countries/regions and 204 cities. In addition to serving as a reflection of a learner’s Japanese language ability, the JLPT is also a vital reference for companies and industries regarding employment, promotion, and qualification. As such, the JLPT stands as the most authoritative and globally influential official examination for Japanese language learners seeking to demonstrate their Japanese language skills. As its scope has expanded through intensified promotion, the JLPT has become increasingly influential worldwide, and more widely recognized, thereby attracting more learners. Thus, the JLPT plays an essential role in promoting Japanese language education and enhancing Japan’s soft power.

This paper presents statistical data on the nations (and regions) and cities that administer the JLPT, as well as the number of examinees over a 10-year period spanning 2012 to 2021. Notably, prior to 2010, the JLPT was conducted once annually, whereas it has since been held twice a year, in July and December, respectively; these biannual exams are also reflected in the statistics, which are tallied separately. It should be noted that the number of examinees is a count of those who actually sat for the exam, not those who registered. Furthermore, the presented data on countries (and regions) and cities where the exams are held, as well as the number of examinees, exclude figures for Japan. These figures give insight into the scope of the Japan Foundation’s efforts to promote the JLPT, and trends in its promotion can be discerned from the data spanning the past decade.

**Figure 4:**
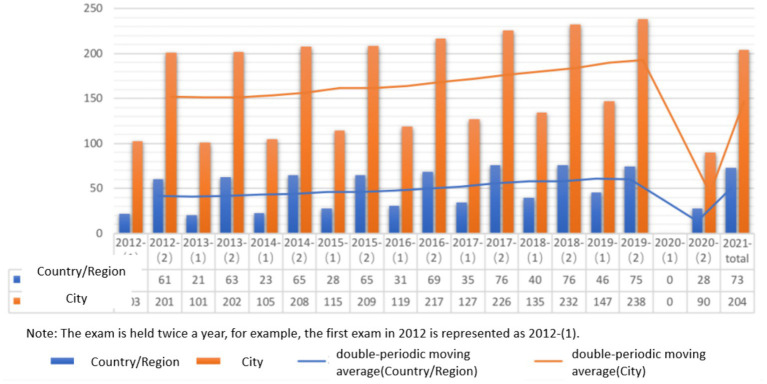
Trend of global promotion of Japanese-Language Proficiency Test (2012–2021).

The findings of this study are presented through the use of bar charts and moving averages (Figure 4), with Table 6 displaying the respective results. It is observed that the scale of the second JLPT exam exceeded that of the first exam every year between 2012 and 2021. The moving average indicates a steady and gradual increase in JLPT exam promotion, reaching its peak in 2019, suggesting that this year witnessed the largest-scale administration of the JLPT. Although the pandemic disrupted the administration of the first JLPT exam in 2020 and substantially reduced the scale of the second exam compared to previous years, the scale has been gradually rising since 2021. It should be acknowledged that the JLPT, being an offline test, has experienced a decline in scale since the global outbreak of COVID-19 in March 2020. However, the analysis of number 1–10 indicates that the Japan Foundation has implemented corresponding measures to mitigate the effects of the pandemic on offline promotion methods, such as examinations, lectures, promotion, and teaching and research activities, after 2020. As indicated below, for instance, through the promotion of Japanese films and the provision of Japanese language teaching resources online, Japan has endeavored to respond to the global pandemic situation and increase the promotion of Japanese culture and language education online.

**Table 6:** Countries and cities where the Japanese-Language Proficiency Test (JLPT) is held worldwide as well as the number of test takers (2012–2021).

Year	Country/Region	City	Number of examinees	Total number of exams
2012	22	103	202,943	449,066
	61	201	246,123	
2013	21	101	198,962	441,244
	63	202	242,282	
2014	23	105	206,961	449,464
	65	208	242,503	
2015	28	115	215,705	468,450
	65	209	252,745	
2016	31	119	227,852	509,664
	69	217	281,812	
2017	35	127	262,930	580,704
	76	226	317,774	
2018	40	135	300,903	644,144
	76	232	343,241	
2019	46	147	347,517	729,450
	75	238	381,933	
2020	—	—	—	181,528
	28	90	181,528	
2021	73	204	315,654	315,654

### Japanese language learning assistance

**Figure 5:**
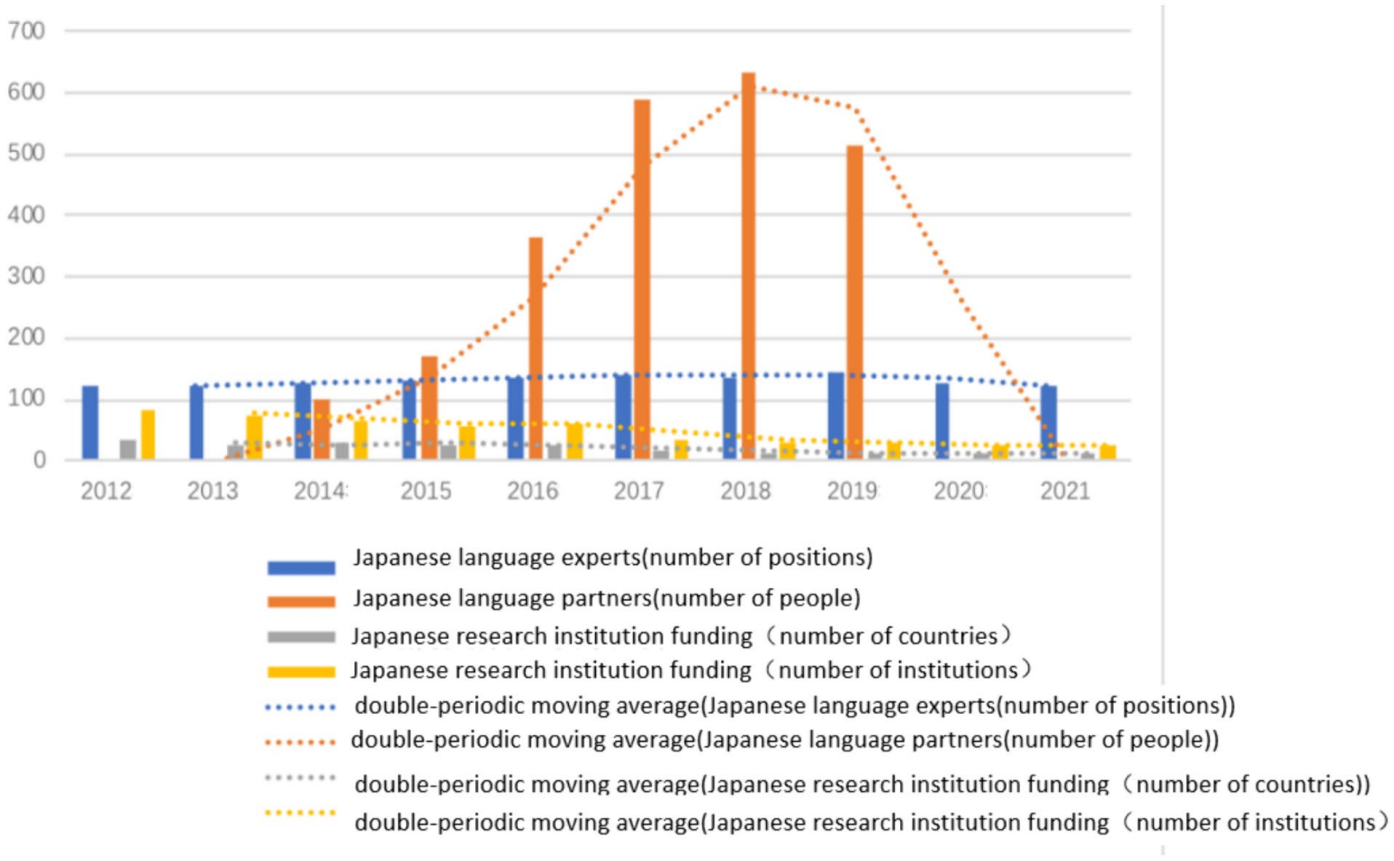
Japanese language experts, Japanese language partners, and Japanese research institution Funding (2012–2021).

The Japan Foundation accords equal priority to fostering cultural affinity with the citizens of target nations and infusing Japanese cultural components into language propagation in its endeavors to promote the Japanese language worldwide. The Foundation’s language promotion program aligns with Japan’s cultural diplomacy strategy. To this end, the Japanese Cabinet approved a preliminary version of the “Comprehensive Economic Measures for a Secure and Growing Future” on December 5, 2019, which earmarked additional funds for the promotion of Japanese language and culture by the Japan Foundation. A significant portion of this allocation has been utilized to deploy specialists and Japanese language study partners overseas. The exports fall into three categories: senior specialists, experts, and assistants who are tasked with supporting Japanese language learning centers abroad and guiding local Japanese language instruction. The learning partners are designated primarily to the Asian region and are essentially volunteers who participate in Japanese cultural activities within and outside of the classroom. The data from previous years indicate that the number of specialists dispatched to other countries remained relatively steady, but experienced a surge from 2012 to 2019, reaching its zenith in 2018, before experiencing a decline in 2020 and 2021 due to the travel restrictions imposed by the pandemic (Table 7). Furthermore, an analysis of the clustered column chart and moving average (Figure 5) reveals that while the number of specialist positions has remained stable, the number of learning partners has increased from 2014 to 2019, culminating in a peak in 2018. The number of nations and institutions that received the funds of Japanese research institutions has gradually declined and has remained low since the 2020 pandemic.

**Table 7:** Japanese language experts, Japanese language partners, and Japanese research institution Funding (2012–2021).

Year	Japanese language experts (number of positions)	Japanese language partners (number of people)	Japanese research institution funding (Number of countries and regions/number of institutions)
2012	123	—	34/82
2013	124	—	26/74
2014	126	100	28/67
2015	133	170	27 / 57
2016	137	364	25/61
2017	140	591	17/35
2018	136	635	13/31
2019	145	515	13/28
2020	126	—	12/25
2021	121	—	12/27

Meanwhile, the initiative to dispatch Japanese language partners was inaugurated at the ASEAN Summit in December 2013 under the auspices of the “Toward Interactive Asia through ‘WA Project’”. The Asia Center, established in Tokyo in April 2014, is entrusted with the task of facilitating Japanese language acquisition and promoting two-way exchanges in arts and culture. According to statistics, over the span of roughly 6 years, from 2014 (when the Center was founded) to 2020, the program has instructed 678,650 learners of Japanese in Asia, dispatched 2,375 Japanese language partners, and hosted or taken part in more than 2,500 bilateral exchange events in the arts and culture, with a total of 5,538,490 participants. Due to the pandemic in 2020, all partners scheduled to be sent to various countries were suspended, and the partner program went entirely virtual in 2020 and 2021 (number 10). As noted in the announcement from the Japan Foundation, the Asia Center, responsible for cultural exchanges in the Asian region, officially closed on March 31, 2022. The newly established “Japanese Language Partners Division” will dispatch experts and partners to other countries, which suggests that the program will not be terminated after 2022 but rather will be administered by a specialized unit that better clarifies the strategic importance of the Japanese language partner business.

Moreover, the analysis indicates that several Japanese language education initiatives were conducted online, which served as an exemplar. For instance, learning partners, who were originally slated to be dispatched to Thailand, delivered a month-long online instruction on pronunciation and sentence construction to students in the target country, garnering widespread public acclaim. As Asia is home to a significant number of Japanese language learners, there is both a geographic advantage and a demographic dividend for various human exchanges and Japanese language education support endeavors. It is noteworthy that, in this process, Japan not only endeavors to promote its language, but also stresses the importance of “two-way” and “collaborative” communication with Asian countries (number 9), which facilitates the advancement of the Japanese language and lays the groundwork for cultural diplomacy by fostering friendship and goodwill among people. More crucially, it also consolidates Japan’s foreign policy in Asia, shapes a positive national image, and contributes to peace and stability among Asian countries. These measures are highly significant for the dissemination of the Japanese language and form a crucial component of Japanese language promotion. The Japan Foundation aligns with the government’s diplomatic policies and emphasizes Japanese studies, international dialogue, and two-way exchanges in the intellectual community, in order to establish a foundation for language spread and the development of soft power through mutual communication and understanding.

### Japanese online teaching resources

**Figure 6:**
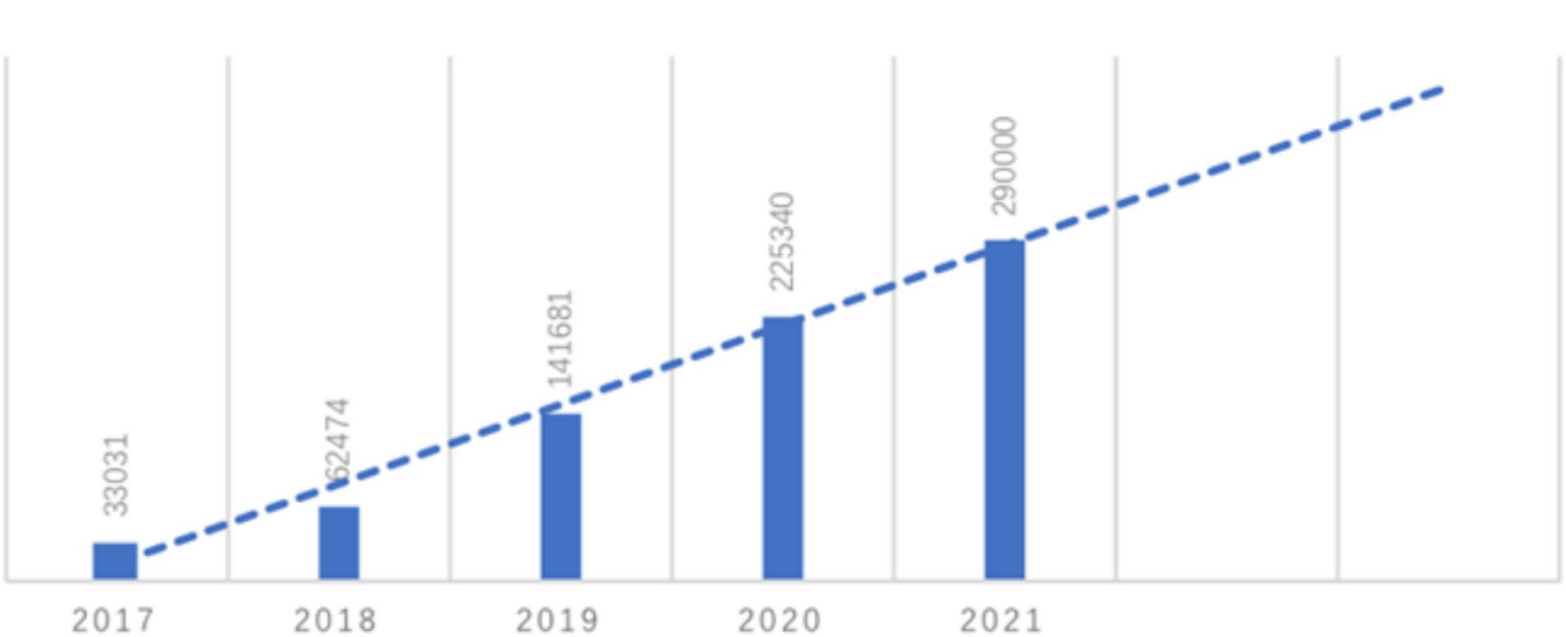
Use of E-learning platform (2017–2020).

The Japan Foundation has prioritized enhancing the domain of e-learning, which is a crucial component of Japanese language education planning. As a pedagogical method, it has been developed with significant efforts since 2020 and adapted to the exigencies of the special period during the global pandemic. An analysis of the number of e-learning platform visitors from 2017 to 2021 (Table 8) indicates a significant year-on-year increase in e-learning platform users, particularly in 2018 and 2019, which can be described as almost exponential growth. Moreover, the linear prediction trend line suggests that the use of e-learning will continue to rise steadily beyond 2021 (Figure 6), reflecting the efficacy of the Japan Foundation’s efforts to create an online teaching platform with positive momentum.

**Table 8:** Use of E-learning platform (2017–2020).

Year	Visitors
2017	33,031
2018	62,474
2019	141,681
2020	225,340
2021	290,000

In terms of the types of online teaching resources for Japanese, there are mainly teaching platforms, teaching materials, content databases and applications for computers and phones. Taking 2020 as an example, the use of online Japanese teaching platforms shows that there are 14 types of popular online teaching resources (Table 9), including the comprehensive online resource platforms, online content databases and applications. In terms of the number of hits or logins, the most influential online textbook is “Marugoto plus” which received more than 9.75 million views in 2020. Online teaching resources provide learners with learning materials and teachers with modern technology and tool-based platforms, which are useful for language teaching and learning and respond to the trend of online education in the post-pandemic era. Japan has focused on the promotion of Japanese language through online teaching platforms and used modern technology as a means and online teaching platforms as a carrier to infinitely expand the geographical space for promotion.

**Table 9:** Use of online teaching resources in 2020.

Name of online teaching resources	Type	Use
JF Japanese e-Learning Website (JFにほんご eラーニング みなと)	Online teaching platform	225,000(logins)
Japanese Textbook Website for Everyone (みんなの教材サイト)	Online teaching platform	1,810,000(views)
Marugoto: Japanese Language and Culture (まるごと 日本のことばと文化)	Online teaching material	6,110,000(views)
Marugoto plus (まるごと +)	Online teaching material	9,750,000(views)
Irodori: Japanese for Life in Japan (いろどり 生活の日本語)	Online teaching material	2,520,000(views)
Hirogaru: Get more of Japan and Japanese (ひろがる もっといろんな日本と日本語)	Online teaching material	780,000(views)
Japanese in Anime & Manga (アニメ・マンガの日本語)	Online teaching material	1,150,000(views)
Nihongo E-na: Portal for Learning Japanese (NIHONGO eな)	Online teaching material	1,370,000(views)
Japanese Care-Navi (日本語でケアナビ)	Online teaching material	900,000(views)
Web Version: Erin’s Challenge! I Can Speak Japanese (ウェブ 版「エリンが挑戦!にほんごできます。」/ 「エリンが挑戦!にほんごできます。」コンテンツライブラリー)	Online teaching material/content library	3,240,000(views)
Challenge with Erin: Japanese Language Test (application for beginners) (エリンと挑戦!にほんごテスト(初学者向けアプリ))	Application	18,000(downloads)
HIRAGANA Memory Hint	Application	90,000(downloads)
KATAKANA Memory Hint	Application	50,000(downloads)
KANJI Memory Hint 1,2&3	Application	80,000(downloads)

### Cultural communication

Language is a social construct that functions as both a product and an essential component of social culture (Halliday, 1978). As a subdivision of sociolinguistics, language planning is not solely linked to language itself, but is rather intertwined with sociology, economics, demography, and education. A crucial objective of language planning is to facilitate the transmission of cultural values. From the perspective of linguistic semiotics, mathematical signs, as well as various art forms, including music, dance, visual arts, and images, can all be regarded as semiotic signs (Halliday, 1992). Among them, film occupies a unique position as a semiotic system and ideographic phenomenon (Metz, 2011). The Japan Foundation endeavors to strengthen the connection between the Japanese language and its users through cultural symbols such as films, publications, translated works, art, and architecture. This objective aligns with Japan’s national strategy of cultural diplomacy and contributes significantly to the development of Japan’s soft power.

Through the analysis of the dissemination of Japanese films and television programs overseas from 2012 to 2021 (refer to Table 10), it is evident that the number of countries in which Japanese films were released, as well as the number of films released, remained stable from 2012 to 2019. In 2014, a record number of 152 Japanese films were released in various countries. Although the number of releases and countries showcasing Japanese movies dropped in 2019 and 2020 due to the pandemic, the promotion of many movies shifted from offline to online platforms or adopted a mixed online and offline approach. To overcome geographical and economic limitations, the Japan Foundation has been developing new online projects in various fields to extend the reach of its works to inaccessible areas. For instance, the video series “STAGE BEYOND BORDERS” aims to disseminate art and culture during the pandemic. In 2019, with stage performances around the world being continuously canceled or postponed, the project aimed to introduce Japanese productions to people worldwide interested in performing arts. The “STAGE BEYOND BORDERS” project promoted a total of 83 productions overseas from 2019 to 2021. Several performances were accompanied by multilingual subtitles in up to 11 languages, receiving over 9.5 million online views and attracting audiences from 111 countries and regions worldwide. In 2021, despite scaled-down artistic exchanges and humanistic dialogues in most countries due to the pandemic, the “STAGE BEYOND BORDERS” project successfully showcased 20 Japanese films to 25 countries in the “Japanese Film Festival Online 2022” with approximately 320,000 viewers. Japan promotes its films, TV dramas, and documentaries overseas to enhance cultural and artistic exchanges, particularly in the Asian cultural sphere, through various communication platforms.

**Table 10:** Japanese film and TV promotion overseas (2012–2021).

Year	Film and television promotion: overseas exchange of Japanese films, TV dramas and documentaries
2012	The Japan Foundation organized 100 Japanese film festivals and screenings in 67 countries and regions and provided financial support for 55 screenings of Japanese films in 25 countries.
2013	The Japan Foundation organized 102 Japanese film festivals and screenings in 70 countries and one region and sponsored 23 Japanese film screenings in 18 countries.
2014	The Japan Foundation organized Japanese film festivals and screenings in 152 cities in 68 countries and regions and sponsored 15 Japanese film screenings in 11 countries.
2015	The Japan Foundation screened 117 Japanese films in 82 countries and regions and financed 19 Japanese film offers in 12 countries and regions. It began broadcasting 31 TV programs in 20 countries worldwide and organized events such as Malaysian Film Week and the Asian International Children’s Film Festival.
2016	The Japan Foundation screened 114 Japanese films in 75 countries and regions and financed 15 films in 23 cities in 15 countries and regions. By the end of 2016, it had broadcast a total of 309 TV programs in 62 countries around the world.
2017	It organized 94 Japanese film festivals and screenings in 67 countries and regions and provided financial support to 11 film projects in 8 countries and regions. It uses the Tokyo International Film Festival as a platform for its film exchange program and launched the JFF (Japanese Film Festival) Asia-Pacific Gateway Initiative.
2018	It organizes Japanese film festivals and screenings in 67 countries and regions and funds 16 film projects in 12 countries and territories. A total of 341 TV programs were broadcast in 53 countries and regions around the world.
2019	It organized Japanese film festivals and screenings in 70 countries and regions and financially supported 19 films in 14 countries and regions. By the end of 2019, it had broadcast 722 Japanese TV programs in 84 countries and regions.
2020	It held Japanese film festivals and screenings in 18 countries and regions and financed 8 films in 7 countries and regions. 583 Japanese TV programs were broadcast in 83 countries and regions around the world.
2021	It conducted Japanese film screenings in a total of 47 countries and regions around the world, while further promoting and expanding new online programs.

The Japan Foundation has designated the era following the pandemic as the “With Corona” era, anticipating that many countries will continue to enforce regular epidemic prevention and control measures, potentially leading to ongoing disruptions in areas such as immigration control and disease prevention policies. In light of these circumstances, adapting to the changing landscape and fostering a blended model of online and offline exchanges in the arts and humanities are essential components of Japan’s cultural diplomacy and soft power strategies.

**Figure 7:**
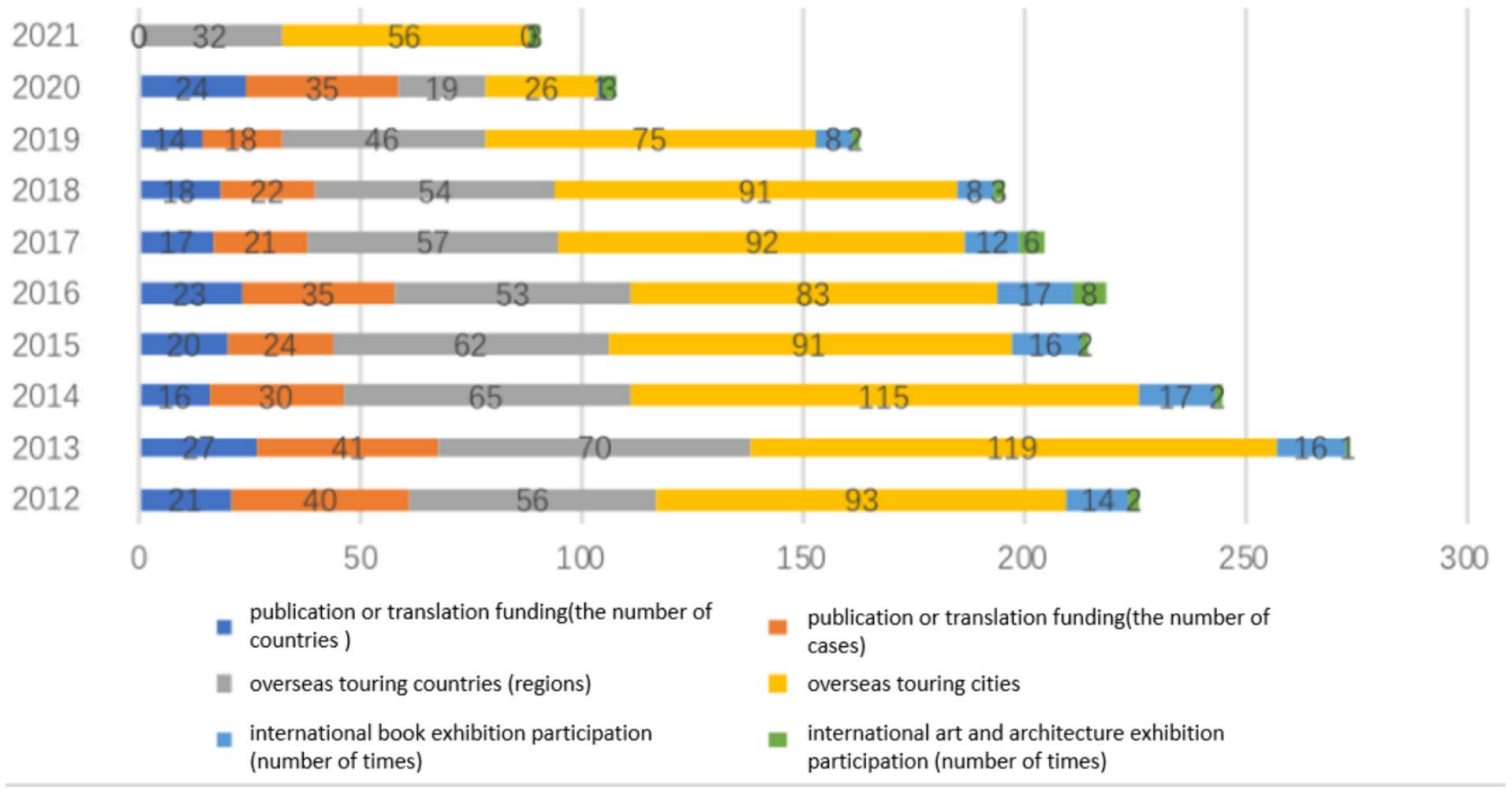
Japan’s cultural and artistic exchanges overseas (2012–2021).

In addition to film and television promotions, Japan has bolstered cultural exchanges with different countries through book exhibitions, publications, translation grants, overseas touring exhibitions, art exhibitions, and architectural exhibitions. This study conducted a comprehensive search of various reports from 2012 to 2021 to collect data related to some of these areas (refer to Table 11). The following six indicators serve as the horizontal coordinates: (1) publication or translation funding countries, (2) number of publication or translation funding cases, (3) overseas touring countries (regions), (4) overseas touring cities, (5) international book exhibition participation (number of times), and (6) international art and architecture exhibition participation (number of times) (refer to Figure 7). A comparison of the six indicators reveals that overseas touring exhibitions have been an area that the Japan Foundation has paid great attention to, as they involve a variety of fields such as design, architecture, photography, crafts, Japanese martial arts, and popular culture to coincide with national diplomatic strategies and important events. Such events not only support Japan’s national diplomatic activities but also increase the influence and appeal of the Tokyo Olympics in 2020. This also proves that cultural and artistic exchanges are powerful approaches and foundations for soft power building. Looking at each indicator separately, the three indicators of publications, translation grants, and overseas touring exhibitions were the highest in 2013, indicating that the promotion of culture and art overseas was stronger and had the widest coverage in 2013. Secondly, in terms of international book exhibitions, the number declined after 2018 and dropped significantly after the pandemic in 2020. As for participation in international art and architecture exhibitions, the number was the highest in 2016, followed by 2017. Overall, Japan’s cultural and art exchanges overseas have been generally steady until 2020, but many events were shifted to online platforms due to restrictions on offline exhibitions in response to the pandemic. However, in 2021, the Japan Foundation still hosted various exhibits offline covering a total of 32 countries and regions and 56 cities through touring exhibitions, such as the Japanese Art Exhibition and the Venice Architecture Biennale (Italy) in Germany and Poland. This shows that the Foundation is still making considerable efforts to exchange and promote culture and art through various offline activities, which indicates that it has not weakened its efforts to build soft power in general, but only shifted some of its efforts online. Thus, tracking online activities is crucial for the future.

**Table 11:** Japan’s cultural and artistic exchanges overseas (2012–2021).

Year	International book exhibition participation (number of times)	Publication or translation funding (the number of countries and cases)	Overseas touring countries (regions)/cities	International art and architecture exhibition participation (number of times)
2012	14	21 countries, 40 cases	56 countries (regions) / 93 cities	2
2013	16	27 countries, 41 cases	70 countries (regions) / 119 cities	1
2014	17	16 countries, 30 cases	65 countries (regions) / 115cities	2
2015	16	20 countries, 24 cases	62 countries (regions) / 91 cities	2
2016	17	23 countries, 35 cases	53 countries (regions) / 83cities	8
2017	12	17 countries, 21 cases	57 countries (regions) / 92 cities	6
2018	8	18 countries, 22 cases	54 countries (regions) / 91 cities	3
2019	8	14 countries, 18 cases	46 countries (regions) / 75 cities	2
2020	1	24 countries, 35 cases	19 countries (regions) / 26 cities	3
2021	—	—	32 countries (regions) / 56 cities	3

In addition, data analysis reveals that there are many overseas communication cases in the plastic arts of Japan, such as handicraft, pottery, sculpture, Japanese dolls, etc. (Due to space limitations, this paper will not go into details). According to *the 2018 Plan of the Japan Foundation*, the Foundation focuses on cultural and artistic exchanges in different regions. In 2018, for example, as for East Asia, on the occasion of the 40th anniversary of the Treaty of Peace and friendship between China and Japan, Japan mainly stepped up movie promotion in China. As for North America, based on changes in Japan-US relations, it holds influential international activities, while as for Central and South America, it makes use of Japanese TV programs to promote the development of cultural undertakings, especially in the regions close to Japan.

Artistic and cultural works, such as television programs, films, architectural designs, art pieces, and translated works, serve as a reflection of a country’s customs, historical narratives, ways of life, and regional cultures, among other elements, thereby embodying its cultural values. As such, these works can be considered symbolic tools for cultural communication. In the process of promoting language, these cultural symbols also have an impact on the ideology of citizens in the receiving country. Within different cultural contexts, these symbols can help overcome cultural barriers and facilitate the learning of the language. Therefore, the core of cultural communication is planning for language prestige, using cultural symbols as carriers, which contributes to language promotion within the broader framework of soft power construction.

### Interaction between language promotion and cultural communication

Based on the specific businesses of the Japan Foundation, this paper conducted a thorough analysis of official reports and other data for a total of 10 years from 2012 to 2021 to show the overall picture of the construction of Japan’s soft power led by the Foundation. Through the analysis, we can see that the Japan Foundation places great value on language promotion while also striving to facilitate cultural communication, specifically by strengthening international dialogues in the fields of art, culture, and architecture. The Foundation has undertaken a series of activities aimed at changing the language choices of citizens in recipient countries, which well illustrates that language promotion and cultural communication are important elements in the construction of soft power.

**Figure 8:**
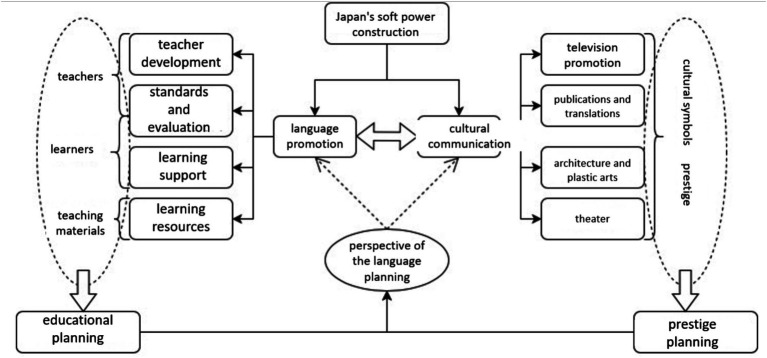
Construction of soft power in Japan.

Moreover, in the realm of soft power, “language” and “culture” operate in tandem, each augmenting the other to establish a foundational pillar of national influence. Measures involving language promotion and cultural communication are essential for constructing an integrated and cohesive framework that synergizes macro-level soft power objectives with micro-level educational and cultural contexts. The strategic alignment of language education planning with language prestige planning becomes crucial. In fact, in the case of Japan, both cultural communication and language promotion are measures of soft power construction that are informed by language planning (Figure 8).

To be specific, language education planning plays a pivotal role in advancing the promotion of the Japanese language. Systematic promotion of the Japanese language, for instance, has increased its visibility and influence, rendering cultural interactions mediated by Japanese more accessible and receptive to a global audience, thereby bolstering Japan’s cultural soft power. Meanwhile, cultural communication, in effect, serves as a form of language prestige planning, enhancing the allure and international standing of the Japanese language. Such enhancements not only broaden the reach of the language but also stimulate enthusiasm among learners. As interest in Japanese culture grows through cultural engagements, more individuals may be compelled to learn the language, further expanding its scope and influence. This reciprocal dynamic creates a positive cycle that promotes the Japanese language and elevates the cultural appeal of Japan, solidifying the groundwork for enhanced soft power.

However, it is critical to maintain a balance between language and cultural promotion efforts. Overextension of these strategies could lead to adverse outcomes such as “cultural conflict” or “cultural hegemony.” From a political standpoint, soft power often involves cultural contestation, where culture becomes a battleground for ideological convergence, as noted by Said (1993). Language promotion, while augmenting a nation’s cultural allure and fostering international communications, may inadvertently foster “language imperialism” (Phillipson, 1992), particularly if a country dominates in political status, linguistic presence, and cultural sway. In the contemporary era, unidirectional export of culture or linguistic hegemony is undesirable. As espoused by the tenets of the Japan Foundation, cultural exchange must be bilateral, aiming ultimately for cross-cultural dialogue and mutual understanding. Thus, language promotion and cultural communication should complement each other, propelling forward the construction and development of a nation’s soft power.

## Discussion

This paper presents a case study on the construction of a state’s soft power. Soft power is commonly referred to as “the third dimension of power” in politics, which denotes the ability of a state to persuade other states to voluntarily accept the same goals in the areas of culture, education, and diplomacy (Luke and Kersel, 2013). Most of the studies on soft power have focused on its role in foreign affairs and enhancing international political status (Glaser and Murphy, 2009; Gallarotti, 2011; Hayden, 2012). Unlike “hard power” and “smart power” (Wilson, 2008), the term “soft” is used to describe “soft power” because it is an inherently integrated, complex, and immeasurable force (Vyas, 2008; Vuving, 2009; Kondo and Muranaka, 2010; Nemoto, 2018) that requires a more systematic and rigorous approach to exploration. Therefore, a theoretical approach to improving national soft power requires quantifying soft power as an implementation content from an interdisciplinary perspective rather than studying it in a single field. This means that conducting case studies is an effective way to analyze the construction of soft power, i.e., to discover concrete ways of implementation through the induction and summarization of experiences before constructing theories.

Many previous studies have examined language promotion institutions in various countries, with particular focus on the Confucius Institutes in China, which are widely regarded as effective tools for China’s soft power construction (Starr, 2009; Li, 2021). Most scholars have analyzed the role of these institutes in China’s public diplomacy policy and examined the influence of cultural and political factors on the construction of soft power, without giving sufficient attention to language education. However, some researchers have recognized the interdependence between soft power and language education, and have proposed a soft power conversion model to explain this relationship (Paradise, 2009; Zhao and Huang, 2010; Rojas, 2021). For instance, Zhu and Li (2014) investigated the role of cultural elements in Chinese language education and examined how China’s geopolitical strategy for language promotion was received and implemented in the UK. By incorporating concepts such as language ideology, attitudes, and motivation into the research of language promotion, Zhu and Li’s study enriched the field of soft power research. Nonetheless, most of the existing literature focuses on a single discipline and examines only one factor in the construction of soft power, thereby failing to establish interdisciplinary connections. There is a pressing need to explore both the “language promotion” and “cultural communication” approaches within the overall framework of soft power construction.

Recognizing this gap, our paper makes two innovative contributions that address these shortcomings. First, theoretically, this paper integrates the concept of language planning into the study of soft power based on case studies of Japan’s soft power efforts. It clarifies the roles and values of language education and prestige planning in the construction of soft power, strategically connecting the two approaches—language promotion and cultural communication—through the lens of language planning. In doing so, it enriches and enhances the theoretical framework underpinning soft power research. Additionally, by connecting policy formulation, language promotion institutions, and language teaching practices, and incorporating the perspectives of policy makers, teachers, and students in the soft power discourse, the study aligns macro-level goals of soft power with micro-level educational contexts, resulting in a more holistic framework that bridges macro and micro perspectives. Second, from a practical standpoint, the research explores and expands upon the specific strategies of prestige planning within the realm of soft power through detailed examinations of initiatives in Japan. For instance, a nation can enhance its linguistic prestige through diverse channels such as television, publishing and translation, architecture and visual arts, theater, etc. so as to construct its soft power. This may offer concrete pathways for the soft power endeavors of other nations.

## Conclusion

The development of soft power is a comprehensive endeavor aimed at fulfilling a nation’s strategic objectives. It is imperative to recognize the significance of both language promotion and cultural communication, with the former being incorporated into language education planning, and the latter into language prestige planning. These aspects must then be considered holistically within the framework of soft power construction.

Based on various reports from the Japan Foundation between 2012 and 2021 (Japan’s Soft Power-Squaring the Cool, 2014; Japan’s Pop-Culture Diplomacy, 2017), this paper presents a comprehensive analysis of Japan’s practice of building soft power through the means of “language promotion” and “cultural communication.” The paper particularly concentrates on the broad framework of Japan’s soft power construction and delineates the ways in which the two approaches, as language planning activities, facilitate soft power building. Consequently, this analysis provides insight into the efficacy and consequences of Japan’s soft power construction in the past decade, and enhances the dialogue between language planning and soft power construction.

In future research endeavors, scholars must acknowledge the pivotal roles that language education planning and language prestige planning play in the fabric of soft power development. It is essential to integrate a diverse array of stakeholders, including policy makers, educators, and learners into the comprehensive assessment of soft power dynamics. Concurrently, there should be a concerted effort to examine an expanded repertoire of country-specific case studies. This would entail the data collection and analysis of more exemplars, providing deeper insights into the dual pathways of language education and prestige planning within the realm of soft power construction, and expanding our understanding of the interplay between language education, prestige planning, and the building of soft power. Such investigations will substantiate and enhance the theoretical framework presented in this paper, further elucidating practices and strategies for soft power augmentation through language promotion efforts and cultural engagement.

## Data availability statement

The original contributions presented in the study are included in the article/supplementary material, further inquiries can be directed to the corresponding author.

## Author contributions

JG: Writing – original draft, Writing – review & editing, Formal analysis, Investigation, Resources. SD: Writing – original draft, Writing – review & editing, Methodology, Supervision.

## References

[ref2] Ang I. Isar Y. R. Mar P. (2015). Cultural diplomacy: beyond the national interest? Int. J. Cult. Policy 21, 365–381. doi: 10.1080/10286632.2015.1042474

[ref3] Baldauf R. B. Jr. (2006). Rearticulating the case for micro language, planning in a language ecology context. Curr. Issues Lang. Plan. 7, 147–170. doi: 10.2167/cilp092.0

[ref5] Cline R. S. (1975). World power assessment: the calculus of strategic drift. Boulder, CO: Westview Press.

[ref6] Cooper R. L. (1989). Language planning and social change. Cambridge: Cambridge University Press.

[ref8] Espinosa J. M. (1977). Inter-American beginnings of US cultural diplomacy: 1936–1948, vol. No. 2. Washington, D.C: Department of State, Bureau of Educational and Cultural Affairs.

[ref9] Finn H. K. (2003). The case for cultural diplomacy: engaging foreign audiences. Foreign Aff. 82, 15–20. doi: 10.2307/20033753

[ref10] Gallarotti G. M. (2011). Soft power: what it is, why it’s important, and the conditions for its effective use. J. Political Power 4, 25–47. doi: 10.1080/2158379X.2011.557886

[ref12] Gienow-Hecht J. C. Donfried M. C. (2010). “Searching for a cultural diplomacy,” in Explorations in culture and international history. Vol. 6. (New York: Berghahn Books), 1–264.

[ref13] Gil J. A. (2017). Soft power and the worldwide promotion of Chinese language learning: The Confucius institute project, vol. 167. Bristol: Multilingual Matters.

[ref14] Glaser B. S. Murphy M. E. (2009). Chinese soft power and its implications for the United States. Serial reports by the center for strategic and international studies. 10–26.

[ref15] Haarmann H. (1990). Language planning in the light of a general theory of language: a methodological framework. Int. J. Sociol. Lang. 1990, 103–126. doi: 10.1515/ijsl.1990.86.103

[ref16] Halliday M. A. K. (1978). Language as social semiotic: the social interpretation of language and meaning. London: Edward Arnold.

[ref17] Halliday M. A. K. (1992). New ways of meaning: the challenge to applied linguistics. Thirty Years Linguist. Evol., 59–95. doi: 10.1075/z.61.09hal

[ref19] Hashimoto K. (2018). “Introduction: why language matters in soft power” in Japanese language and soft power in Asia. ed. Hashimoto K. (Singapore: Palgrave Macmillan), 1–12.

[ref20] Haugen E. (1983). The implementation of corpus planning: theory and practice. Prog. Lang. Plan. Int. Perspect. 31, 269–290,

[ref21] Hayden C. (2012). The rhetoric of soft power: public diplomacy in global contexts. Lanham, Maryland: Lexington Books.

[ref22] Held D. McGrew A. Goldblatt D. Perraton J. (2000). “Global transformations: politics, economics and culture,’’ in Politics at the edge. Political studies association yearbook series. Eds. C. Pierson and S. Tormey (London: Palgrave Macmillan).

[ref23] Henne P. S. (2022). What we talk about when we talk about soft power. Int. Stud. Perspect. 23, 94–111. doi: 10.1093/isp/ekab007

[ref25] Iskhakova L. F. (2021). Language learning as a source of soft power: survey result. Yekaterinburg, Russia. Ural Federal University: Стратегии развития социальных общностей, институтов и территорий.—Т. 2.—Екатеринбург, *Vol. 2*. 103–106.

[ref26] Japan’s Pop-Culture Diplomacy (2017). Available at: https://www.mofa.go.jp/policy/culture/exchange/pop/index.html

[ref27] Japan’s Soft Power-Squaring the Cool (2014). Available at: https://www.economist.com/blogs/banyan/2014/06/japans-soft-power.

[ref29] Kondo Y. Muranaka M. (2010). 日本のポップカルチャーファンは潜在的日本語学習者といえるか [Can Japanese pop culture enthusiasts be considered as potential Japanese language learners?]. 国際交流基金日本語教育紀要 [The Japan foundation Japanese-language education bulletin], vol. 6, 1–7.

[ref30] Li S. (2021). China’s Confucius Institute in Africa: a different story? Int. J. Compar. Educ. Dev. 23, 353–366. doi: 10.1108/IJCED-02-2021-0014

[ref31] Luke C. Kersel M. (2013). US cultural diplomacy and archaeology: Soft power, hard heritage. New York: Routledge.

[ref32] Lukes S. (2005). Power: a radical view. 2nd Edn. New York: Palgrave Macmillan.

[ref33] Metz C. (2011). Language and cinema. Berlin, Boston: De Gruyter Mouton.

[ref34] Morgenthau H. (1946). Scientific man versus power politics. Chicago, IL: University of Chicago Press.

[ref36] Nemoto A. (2018). “Japanese pop culture as a motivating factor for Japanese language learners in Qatar,’’ in Japanese language and soft power in Asia. Ed. K. Hashimoto (Singapore: Palgrave Macmillan).

[ref38] Noack C. (2022). “Introduction language and culture in Russia’s soft power toolbox” in Politics of the Russian language beyond Russia. Ed. C. Noack (Edinburgh: Edinburgh University Press), 1–18.

[ref39] Nye J. S. (1990). Soft power. Foreign Policy 80, 153–171. doi: 10.2307/1148580

[ref40] Nye J. S. (2004). Soft power: the means to success in world politics. New York: Public Affairs.

[ref41] Nye J. S. (2011). Power and foreign policy. J. Political Power 4, 9–24. doi: 10.1080/2158379X.2011.555960

[ref42] Paradise J. F. (2009). China and international harmony: the role of Confucius Institutes in Bolstering Beijing’s soft power. Asian Surv. 49, 647–669. doi: 10.1525/as.2009.49.4.647

[ref43] Paschalidis G. (2009). Exporting national culture: histories of cultural institutes abroad. Int. J. Cult. Policy 15, 275–289. doi: 10.1080/10286630902811148

[ref44] Phillipson R. (1992). Linguistic imperialism. London: Oxford University Press.

[ref45] Repnikova M. (2022). Chinese soft power (Series of Elements in Global China). Cambridge: Cambridge University Press.

[ref46] Rojas I. C. (2021). Rethinking the discursive relevance of pop culture in Japan’s soft power in Chile: a perspective from Japanese language education. Electr. J. Contemp. Jpn. Stud. 21.

[ref47] Roselle L. Miskimmon A. O’Loughlin B. (2014). Strategic narrative: a new means to understand soft power. Media War Conflict 7, 70–84. doi: 10.1177/1750635213516696

[ref48] Said E. W. (1993). Cultural and imperialism: 1st vintage book. New York: Knopf.

[ref49] Signitzer B. (2013). Public relations and public diplomacy: some conceptual explorations. Benno Signitzer: Von erlebbarem Wissen und Verwissenschaftlichung, 289–306.

[ref51] Starr D. (2009). Chinese language education in Europe: the Confucius institutes. Eur. J. Educ. 44, 65–82. doi: 10.1111/j.1465-3435.2008.01371.x

[ref52] Vuving A. (2009). How soft power works. SSRN Electr. J. 1466220. doi: 10.2139/ssrn.1466220

[ref53] Vyas U. (2008). The Japan Foundation in China: an agent of Japan's soft power? Electr. J. Contemp. Jpn. Stud.,

[ref54] Wilson E. J. III (2008). Hard power, soft power, smart power. Ann. Am. Acad. Pol. Soc. Sci. 616, 110–124. doi: 10.1177/0002716207312618

[ref55] Zhao H. Huang J. (2010). China’s policy of Chinese as a foreign language and the use of overseas Confucius institutes. Educ. Res. Policy Prac. 9, 127–142. doi: 10.1007/s10671-009-9078-1

[ref56] Zhu H. Li W. (2014). Geopolitics and the changing hierarchies of the Chinese language: implications for policy and practice of Chinese language teaching in Britain. Mod. Lang. J. 98, 326–339. doi: 10.1111/j.1540-4781.2014.12064.x

